# Comparison of habitual physical activity in French Bulldogs, Pugs and normocephalic dogs by accelerometry

**DOI:** 10.1017/awf.2023.80

**Published:** 2023-09-11

**Authors:** Mimma Aromaa, Heikki Putro, Liisa Lilja-Maula, Minna M Rajamäki

**Affiliations:** 1 Department of Equine and Small Animal Medicine, Faculty of Veterinary Medicine, PO Box 57, FI-00014 University of Helsinki, Finland; 2 Veterinary surgery Eläinlääkäriasema HauMau, Hietaniemenkatu 7, FI-00100 Helsinki, Finland

**Keywords:** accelerometer, animal welfare, brachycephalic obstructive airway syndrome, French Bulldog, physical activity, Pug

## Abstract

Brachycephalic obstructive airway syndrome (BOAS) is a major welfare concern in flat-faced dog breeds. As BOAS causes respiratory difficulties and exercise intolerance, it can reduce dogs’ daily quality of life (QOL). However, evaluation of QOL in dogs is difficult, and many owners perceive BOAS signs as ‘normal’ for the breed. Accelerometers that measure frequency, duration and intensity of activities can offer an objective way of evaluating dogs’ daily activity and thereby deliver potential insights into QOL. The aim of this study was to assess habitual physical activity of 48 brachycephalic and 23 non-brachycephalic dogs using accelerometers. The accelerometers were used for one week and owners filled in a questionnaire regarding their dog’s well-being and activities. Veterinary-assessed BOAS grading for brachycephalic dogs was determined. Compared with controls, more severely affected French Bulldogs and Pugs had significantly lower total activity counts and spent less time in high activity. In Pugs, mildly affected dogs were also less active, but age can be a contributing factor here, as older age decreased activity in Pugs and controls showed a wider age range. In French Bulldogs, those dogs with no or mild signs of BOAS did not differ from controls regarding their daily activity. In conclusion, accelerometers were easy to use for objective measurement of daily activity in bracycephalic dogs, although a degree of discomfort due to the collar was reported. Results showed that BOAS signs were associated with decreased habitual physical activity. These findings emphasise the importance of actions taken to reduce incidence of BOAS in brachycephalic breeds.

## Introduction

Habitual physical activity is compromised in many chronic diseases, and it has been widely used in human research as an indicator of quality of life (QOL) and as a predictor of treatment effects (Corder *et al.* 2008; Reilly *et al.* 2008; Troiano *et al.* 2008; de Vries *et al.* 2009). QOL in dogs is most commonly evaluated via subjective methods such as questionnaires, including owner assessment of pet’s level of activity among other features of daily life or by veterinary assessment (Slater *et al.* 1995; Robertson 2003; Wojciechowska & Hewson 2005; Councier *et al.* 2010). However, these methods are subjective and may not therefore widely reflect the aspects of daily life at home. Activity monitors can offer a more objective way of evaluating habitual physical activity in animals in their home environment and may provide new insights into daily QOL.

Accelerometers measuring frequency, duration and intensity of activity can be used to quantify daily physical activity by mean activity counts. In the last decade, evaluation of mobility and activity at home has been shown to be a valuable tool in veterinary research (Dow *et al.* 2009; Michel & Brown 2011; Yam *et al.* 2011). Accelerometers have been used in several studies to assess effects of chronic diseases (e.g. orthopaedic and dermatological) or obesity on physical activity in dogs (Nuttall & McEwan 2006; Brown *et al.* 2010b; Morrison *et al.* 2013; Wernimont *et al.* 2018).

Actical^®^ (Mini Mitter Inc, Bend, OR, USA) is an omnidirectional accelerometer designed for research purposes that has been validated for use in dogs to monitor spontaneous activity at home (Hansen *et al.* 2007). It is well-tolerated, non-invasive and can be attached ventrally to the neck with its own collar. Benefits of Actical^®^ include that it can be used to differentiate varying physical activity intensities such as sedentary, light or vigorous activity (Michel & Brown 2011). For the most reliable assessment of a dog’s overall activity, a one-week measuring period has been recommended (Yam *et al.* 2011).

The popularity of brachycephalic, i.e. flat-faced, breeds has soared due to social influences despite their well-known and widely discussed health problems (Ghirlanda *et al.* 2014; Packer & Farnsworth? 2017; O’Neill *et al.* 2018). In recent years the welfare of brachycephalic dog breeds has become a major animal health concern with animal welfare authorities in a number of countries laying down strict regulations as regards breeding of these dogs (Dutch Ministry of Agriculture, Nature and Food Quality 2019; Ross 2022). Brachycephalic breeds suffer from respiratory difficulties caused by brachycephalic obstructive airway syndrome (BOAS), which is directly linked to the anatomical structure of their skull (Oechtering 2010; Emmerson 2014). As exercise intolerance is one major consequence of BOAS, exercise tests and clinical veterinary assessment before and after exercise challenge have been used to assess severity of BOAS (Liu *et al.* 2015; Lilja-Maula *et al.* 2017; Riggs *et al.* 2017; Aromaa *et al.* 2019, 2021). Results of an owner questionnaire for severely BOAS-affected dogs has shown marked restrictions on the daily exercise habits of these dogs (Roedler *et al.* 2013). Owners, however, tend to underestimate the signs caused by BOAS, instead seeing them as a breed feature (Packer *et al.* 2012, 2019; Roedler *et al.* 2013). Therefore, owner questionnaires might give misleading information on QOL and especially on exercise habits and activity. However, veterinary assessment of BOAS severity is important when evaluating QOL in these dogs. Although in our recent studies (Aromaa *et al* 2019, 2021) the majority of young French Bulldogs and Pugs did not have clinically significant BOAS signs, many brachycephalic dogs may even suffer from life-threatening signs, severely compromising their daily QOL (Riecks *et al.* 2007; Roedler *et al.* 2013). Severe BOAS signs, such as pronounced upper respiratory sounds, dyspnoea, sleeping and eating difficulties (Hendricks 2004; Poncet *et al.* 2005; Riecks *et al.* 2007; Roedler *et al.* 2013), have been well documented, but more objective information about QOL in mildly affected brachycephalic dogs at home is needed. Measuring daily habitual activity at home using an accelerometer can therefore offer us a new, improved understanding of the effects of BOAS on dogs’ day-to-day lives.

The aims of this study were to study daily habitual physical activity of French Bulldogs and Pugs with different severity of BOAS signs in their home environment and to compare the results with same-sized non-brachycephalic dogs by using an activity monitor.

## Materials and methods

### Ethical approval

All dogs were privately owned companion dogs whose owners were willing to participate in the study and considered their dogs to be in good health. All owners signed an informed consent. The study protocol was approved by the University of Helsinki Viikki Campus Research Ethics Committee (8/2017).

### Study animals

A total of 48 brachycephalic and 23 non-brachycephalic dogs were enrolled in the study between October 2017 and December 2020. The majority of brachycephalic dogs (33/48) were recruited from our previous study (Aromaa *et al.* 2019) performed at the Veterinary Teaching Hospital, University of Helsinki, Finland. The rest of the brachycephalic dogs were recruited among those participating in the Finnish Kennel Club health screening tests meant for brachycephalic dogs (12/48) and from dogs that later underwent surgical treatment of BOAS at a private veterinary surgery in Helsinki, Finland (3/48). Non-brachycephalic healthy pet dogs in the same age range from among small- or medium-sized breeds were recruited as control dogs. Inclusion criteria for brachycephalic dogs were purebred French Bulldog or Pug, age over 1.5 years and no history of airway surgery due to BOAS. Dogs were excluded from the study if they were pregnant, lactating, discontinued activity measurement prematurely or they had signs that could affect the accuracy of activity measurements, such as current orthopaedic or dermatological condition. All dogs lived in suburban environments.

### Veterinary-assessed BOAS grade

All brachycephalic dogs, except those scheduled for surgery, had veterinary-assessed BOAS grade assigned by the author (MA). This veterinary-assessed BOAS grading (0 = no, 1 = mild, 2 = moderate, 3 = severe signs) was determined by assessing audible upper respiratory sounds and the presence of respiratory effort, dyspnoea and cyanosis before and after exercise, as described in our previous studies (Lilja-Maula *et al.* 2017; Aromaa *et al.* 2019) and based on the study by Liu *et al.* (2015). The dogs were further divided into two classes; no or mild signs of BOAS were considered to be BOAS negative (BOAS–) and moderate or severe signs BOAS positive (BOAS+) (Liu *et al.* 2015). Exercise tests were not performed due patient safety on dogs seeking surgical treatment for BOAS and considered directly to be BOAS+. All control dogs represented non-brachycephalic breeds, and therefore, no BOAS evaluation was performed on them.

### Setting for accelerometer activity levels

The omnidirectional Actical^®^ accelerometer continuously measures the intensity, frequency and duration of movement. The device generates a voltage when it detects a change in acceleration. The voltage is converted to a digital value and compared to the baseline value formed by continuous effects (e.g. gravity) on acceleration. The difference is converted by the associated computer software to a value for the measurement period (epoch) which is further reported as an activity count. (Hansen *et al.* 2007; Belda *et al.* 2018). Cut-off points for the Actical^®^ accelerometer readings for sedentary (staying still, slight movement) and high (at least trotting) activity with 1-min epoch length were collected under supervision from ten French Bulldogs and one control dog (Australian Terrier) at the Veterinary Teaching Hospital, University of Helsinki. The median age for these dogs was 4 (range 1–8) years. Dogs were assessed along a 60-m straight corridor on a leash. Dogs wore an activity collar while staying still or moving slightly (i.e. sedentary activity) and during trotting (i.e. high activity) back and forth in the corridor. In total, 100 epochs for sedentary and 117 epochs for high activity were collected. The upper limit for sedentary activity and the lower limit for high activity was defined as mean (± 2SD) and moderate activity as values between these limits. As such, established upper activity count limit for sedentary activity was 347 (111 [± 118]; range 0–427) and lower limit for high activity 1,343 (mean 4,915 [± 1,786; range 1,064–9,637).

### Owner questionnaire and activity diary data

Owners filled a questionnaire regarding their dogs overall well-being, any medications, activity device use, alternating events during the study week and exercise habits. Additionally, more specific questions about respiratory, gastrointestinal and dermatological signs were posed. An activity diary (24 h divided into 30-min slots) was completed during the measurement period in which owners were asked to describe briefly their dog’s activities during each day (e.g. sleeping, walking, home alone). Both questionnaire and activity diary were designed by authors. All owners were instructed how to fill the diary and provided with a model. The questionnaire and activity diary model for owners can be seen in the Supplementary material.

### Accelerometer data collection and analysis

The habitual physical activity in dogs was measured using Actical^®^ accelerometer. Fourteen accelerometers were available and randomly distributed to study dogs. The Actical^®^, encased in a protective metallic sheath, was attached with its own collar to each dog and all 14 accelerometers were used evenly in all study groups. Owners were instructed to remove the device only during water activities and not to attach a walking leash to the same collar. Measurements were not collected during summer months. The accelerometer was set to a 1-min epoch length and held continuously for seven days. After the measurement period, the data were downloaded from the accelerometer with the Actical^®^ reader device. The activity value was converted and reported as activity count number for each minute in the measurement period. Total activity count of the measurement period was converted to mean total activity count per day to maintain comparability if the owner had removed the collar for a few hours before the end of the full seven-day period.

### Statistical analysis

Descriptive statistics are presented as means (± SD) for continuous normally distributed variables or as a median and range for non-continuous or non-normally distributed variables. The normality of the activity data was assessed using the D’Agostino-Pearson test of normality. Statistical comparisons between groups were performed with an ANOVA method, and Tukey’s correction was used in comparisons. Multiple linear regression analysis was performed to assess relationships between potential correlates (age, sex and BOAS class) and outcome variables (proportion of time spent in sedentary, moderate and high activity in the measurement period) of controls, French Bulldogs and Pugs. To meet the assumptions of linear regression analysis, log-transformation was carried out on all outcome variables prior to analysis. *P*-values < 0.05 were considered significant. Statistical analyses were conducted using GraphPad Prism Mac 9.3.1^®^

## Results

### Signalment and BOAS grading

The demographics of the dogs are presented in Table 1. The brachycephalic population consisted of 25 French Bulldogs (12 BOAS–, 13 BOAS+) and 23 Pugs (12 BOAS–, 11 BOAS+). The distribution of the veterinary-assessed BOAS grades of the brachycephalic dogs is presented in Table 2. The 24 control dogs included 14 different breeds: six Cairn Terriers, four standard Dachshunds, two Mittelspitzs, two mixed breeds and one of each of the following: Jack Russell Terrier, Cocker Spaniel, Welsh Terrier, Swedish Vallhund, Danish-Swedish Farmdog, Smooth Fox Terrier, Kromfohrländer, Shetland Sheepdog, Australian Terrier and Schapendoes.

**Table 1.** Demographic data for study dogs

	French Bulldogs (n = 25)		Pugs (n = 23)		
	BOAS–	BOAS+	BOAS–	BOAS	Control dogs (n = 24)
Sex					
Female	9	6	8	8	14
Male	3	7	4	3	10
Age (years; median with range)	3.0	3.5	5.5	6.5	3.3
	(2–5)	(1.5–6.5)	(2.5–8)	(2.0–9.5)	(1.5–7)
Weight (kg; mean [± SD])	12.5	13.3	8.8	8.4	9.1
	(± 1.7)	(± 1.6)	(± 1.2)	(± 1.5)	(± 2.0)

SD, standard deviation; BOAS, brachycephalic obstructive airway syndrome; BOAS–, no or mild signs BOAS+, moderate or severe signs.

**Table 2.** Distribution of veterinary-assessed brachycephalic obstructive airway syndrome (BOAS) severity grade in French Bulldogs and Pugs

	French Bulldogs (n = 25)	Pugs (n = 23)
BOAS grade		
0	8	2
1	4	10
2	8	7
3	5	4

BOAS grade, 0 = no signs, 1 = mild, 2 = moderate, 3 = severe; light grey=BOAS– ; dark grey = BOAS+

### Owner questionnaire and activity diary

None of the owners reported that the questionnaire or diary was difficult to complete. Only one Pug owner (1/23) reported that respiratory signs were limiting the daily activity of the dog. Four of the French Bulldogs (4/25; 16%) had persistent dermatological problems. Four of the French Bulldogs (4/25; 16%) and two of the Pugs (2/23; 9%) had mild gastrointestinal signs (i.e. vomiting and regurgitation) during the measurement period. Four of the French Bulldogs (4/25; 16%) were on oclacitinib medication for atopy, and one Pug (1/23, 4%) was on phenobarbital medication for epilepsy. All control dog owners considered their dogs to be in good health, and none were using any medications. Multi-dog households were equally common among the study groups (16/25 FBs [64%;] 15/23 Pugs [65%]; 15/24 controls [63%]). During the measurement period 11/25 French Bulldog (44%), 13/23 Pug (57%) and 12/24 control dog owners (50%) reported some specific scenarios affecting their dog’s activity levels such as vacations, weather, owner’s personal reasons, home alone time or taken care of by pet sitters. Altogether, 4/25 French Bulldog (16%), 6/23 Pug (26%) and 1/24 control dog owners (4%) described the activity collar as somewhat uncomfortable for their dog during the measurement period, but only one French Bulldog owner discontinued the measurement period as a result of this. According to activity diaries and owners’ descriptions of dogs’ exercise habits, all dogs were exercising under their owners’ supervision. None of the dogs were predominantly free-roaming or lived in a kennel environment. Descriptive data of outdoor activities are presented in Table 3.

**Table 3.** Owner-reported descriptive data of outdoor activity of dogs

	French Bulldogs (n = 24)*	Pugs (n = 23)	Control dogs (n = 24)
Dog walking always on leash	15 (63%)	14 (61%)	16 (67%)
Dog walking always unleashed	1 (4%)	3 (13%)	3 (13%)
Dog walking on leash and unleashed	9 (38%)	4 (17%)	5 (21%)
Opportunity to go outdoors unleashed several times a day	11 (46%)	11 (48%)	18 (75%)

* One French Bulldog owner did not answer questions regarding the dog’s outdoor activities.

### Activity measurement results

**Figure 1.**
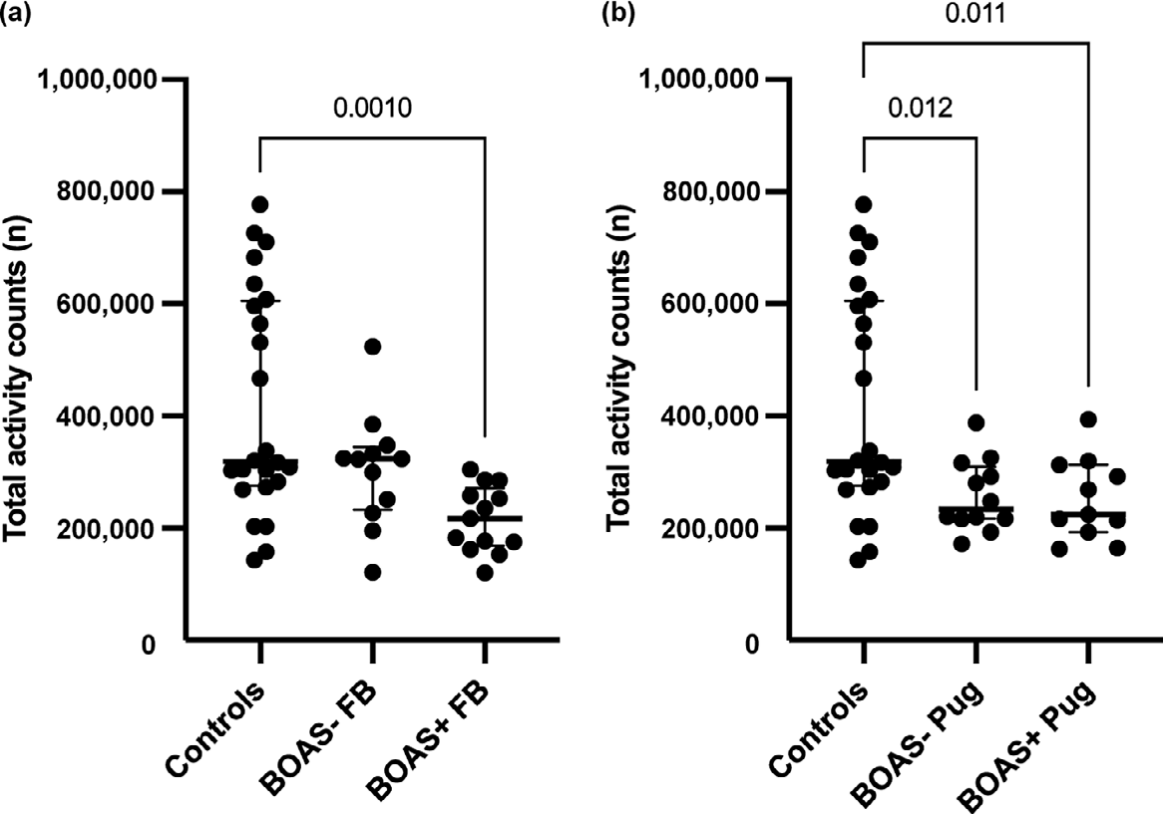
Boxplots of mean total daily activity counts for brachycephalic obstructive airway syndrome (BOAS) positive (+) and negative (–) (a) French Bulldogs (FB) and (b) Pugs compared with controls with median +/- IQR. Significant differences (*P* < 0.05) between groups are marked.

Twelve owners removed the collar for a few hours prior to the end of the seven-day measurement period. Only one data collection failed due to a problem with the accelerometer itself. BOAS+ French Bulldogs (*P* = 0.0010) and both BOAS+ (*P* = 0.011) and BOAS– Pugs (*P* = 0.012) had significantly lower mean total activity counts per day than control dogs. The comparisons between BOAS– and BOAS + groups by breed and control group are shown in Figure 1.

**Figure 2.**
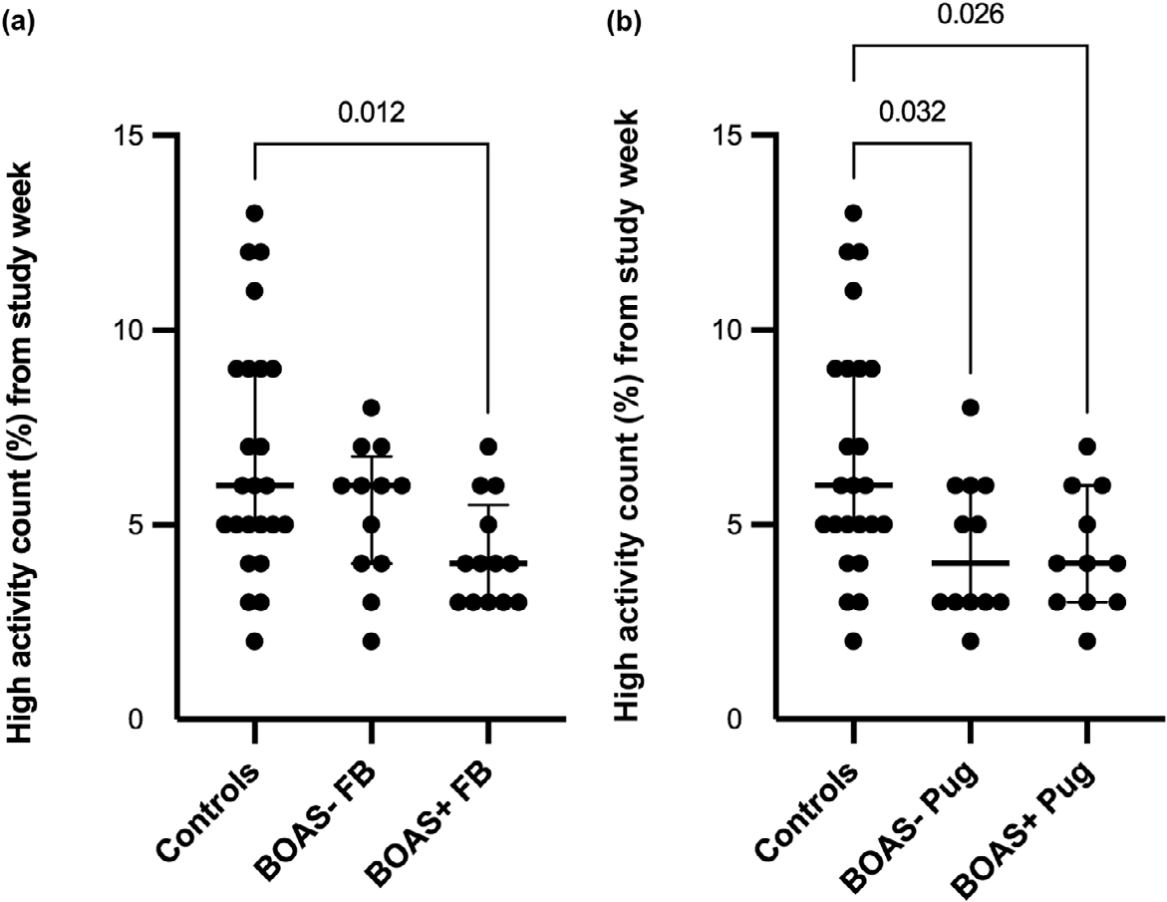
Comparisons of proportion of time spent in high activity during the measurement period between the (a) BOAS– and (b) BOAS+ groups by breed and control group with median +/- IQR. Significant differences (*P* < 0.05) between groups are marked.

**Figure 3.**
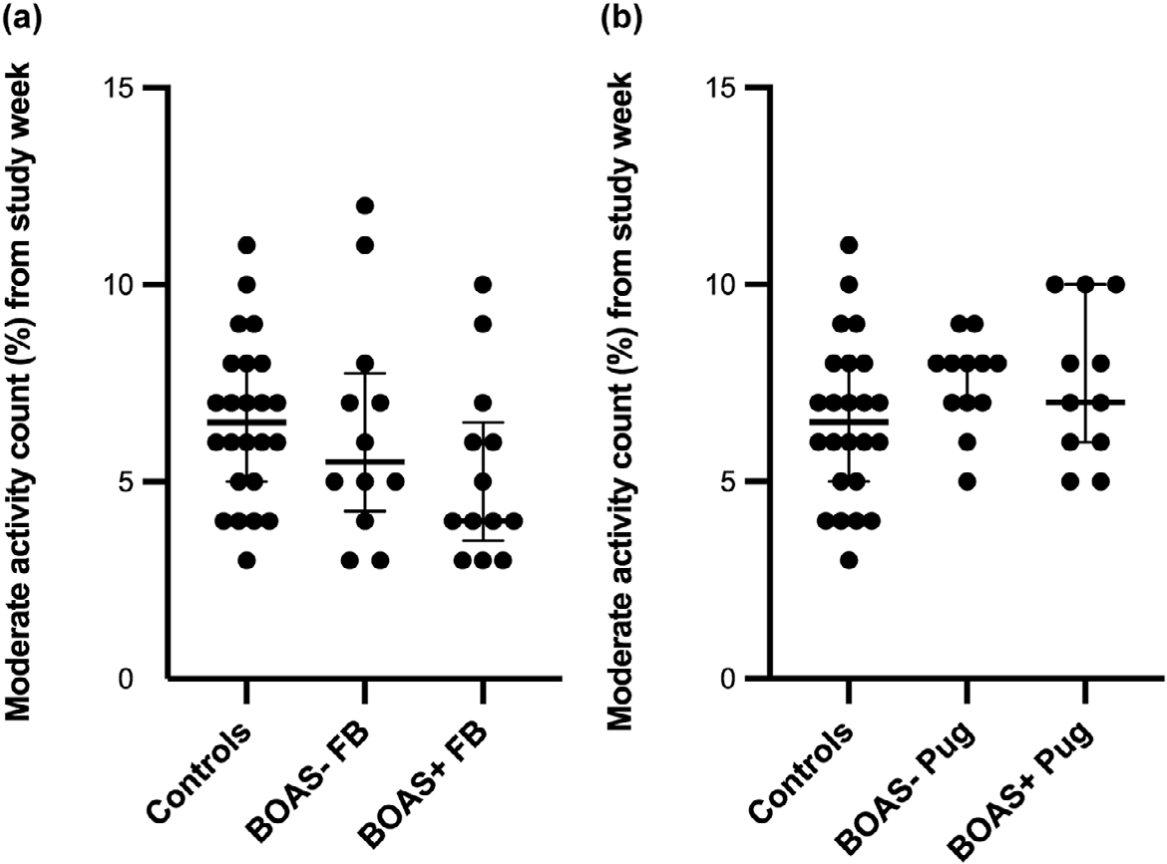
Comparisons of proportion of time spent in moderate activity during the measurement period between the (a) BOAS– and (b) BOAS+ groups by breed and control group with median +/- IQR. Significant differences (*P* < 0.05) between groups are marked.

**Figure 4.**
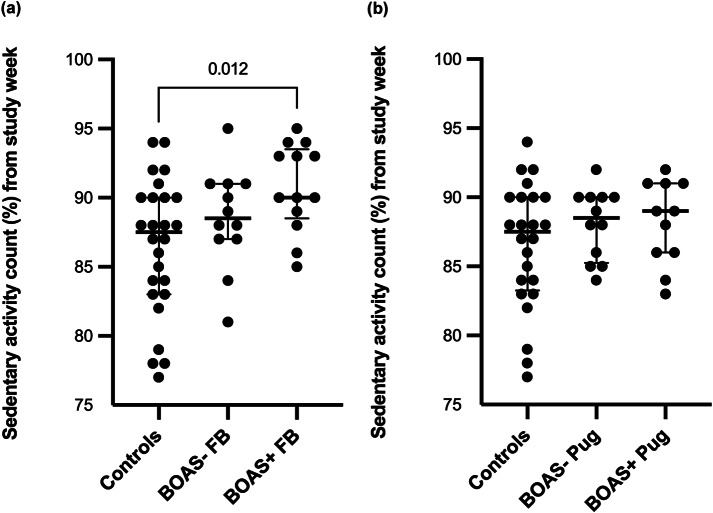
Comparisons of proportion of time spent in sedentary activity during the measurement period between the BOAS– and BOAS+ groups by breed and control group with median +/- IQR. Significant differences (*P* < 0.05) between groups are marked.

BOAS+ French Bulldogs (*P* = 0.012) and both BOAS+ (*P* = 0.026) and BOAS– Pugs (*P* = 0.032) spent significantly less time in high activity during measurement period when compared to the control group. Additionally, BOAS+ French Bulldogs (*P* = 0.012) spent significantly more time in sedentary activity compared to control dogs. The comparisons of proportion of time spent in sedentary, moderate and high activities during the measurement period between the BOAS– and BOAS+ groups by breed and control group are presented in Figures 2, 3 and 4.

To assess the potential impact of atopy and epilepsy on activity results, additional analyses were carried out. Significance in total activity between BOAS+ French Bulldogs and controls (*P* = 0.005) remained when dogs on oclacitinib medication were removed. Additionally, removing one BOAS– Pug with phenobarbital medication did not affect significance level in total activity between BOAS– pugs and controls (*P* = 0.017). Similarly, no effect on significance levels between BOAS+ French Bulldogs and controls were seen in high (*P* = 0.044) or sedentary (*P* = 0.049) activity. Also, significance levels for high activity remained between BOAS– pugs and controls (*P* = 0.036).

Although owners of control dogs reported that their dogs had more opportunities than brachycephalic dogs to venture outside unleashed (Table 3), comparing diary-marked activity to activity counts showed that only a minority (median 16%, IQR 13%, range 0–70%) of high activity periods in these 18/24 control dogs were linked to these opportunities. In 14/18 of these control dogs, a maximum 20% of high activity was linked to the opportunity to venture outdoors unleashed. From the remaining 4/18, one dog had majority (70%) of high activity linked to time spent unleashed in stables with owner and it also had the highest total and high activity counts. Three of these dogs were below the median for their total and high activity counts. Removing this one control dog with highest activity reading due to long-lasting unleashed outdoor time did not alter statistical significance between groups. The results of the comparisons of mean total activity counts after described exclusion between BOAS– and BOAS+ groups by breed and control group are able to viewed as Supplementary material Figure 1. Additionally, four control dogs that had most opportunities to go outside unleashed are highlighted in the figure to provide insight into their total activity levels.

In multiple linear regression analysis for all activity classes, age alone was significant in controls (sedentary: *P =* 0.0021, *R*² 44%; moderate: *P =* 0.024, *R*² 26%; high: *P* = 0.011, *R*² 29%) and Pugs (sedentary: *P* < 0.001, *R*² 48%; moderate: *P =* 0.0037, *R*² 37%; high: *P =* 0.031, *R*² 22%). For every one year increase in age, there was a 2% (CI 1–3%) increase for controls and a 1% (CI 0–2%) increase for Pugs in sedentary activity. Similarly, we observed an 8% (CI 1–14%) decrease for controls and a 7% (CI 2–10%) decrease for Pugs in moderate activity and a 13% (CI 3–21%) decrease for controls and a 9% (CI 1–17%) decrease for Pugs in high activity. In French Bulldogs, there were no significant correlates in any activity class.

Descriptive data on distribution of total activity counts regarding age, sex and weight in French Bulldogs and Pugs are added as Supplementary material Figure 2.

## Discussion

As objective evaluation of QOL is difficult in animals, activity measurements can act as an additional tool providing greater insight into QOL. Our study showed that BOAS+ French Bulldogs and both BOAS+ and BOAS– Pugs had significantly lower total activity counts and spent less time in high activity than controls. However, in controls and Pugs activity was observed to decrease with age, in line with previous studies, and dogs spent most of their time in sedentary activity (Michel & Brown 2011; Morrison *et al.* 2014). Although BOAS has been shown to cause exercise intolerance during submaximal exercise testing (Liu *et al.* 2015; Lilja-Maula *et al.* 2017; Aromaa *et al.* 2019, 2021; Villedieu *et al.* 2019), to our knowledge, this the first study to assess total habitual physical activity and activity levels in brachycephalic dogs using accelemetry in the home environment.

Actical^®^ accelerometer has been validated in dogs and used to measure total activity and differentiate physical activity intensities in earlier studies (Hansen *et al.* 2007; Michel & Brown 2011). Actical^®^ accelerometers should be used either within their own protective shell or without it and not be attached to the same collar as the walking leash, as this can affect measurement results (Martin *et al.* 2017). Although some inter-device variability in Actical^®^ activity counts has been reported, a single device has not been shown to consistently over- or underestimate the activity counts (Olsen *et al.* 2016). Due to size differences, activity counts can differ between breeds (Brown *et al.* 2010a; Morrison *et al.* 2014). In previous studies, the majority of dogs have weighed over 10 kg (Hansen *et al.* 2007; Michel & Brown 2011). We therefore reassessed the thresholds for sedentary, moderate and high activites in dogs meeting our study inclusion criteria. The accelerometer epoch length has varied from 15 s to 1 min in previous studies assessing companion dogs’ activities (Michel & Brown 2011; Morrison *et al.* 2013, 2014; Yashari *et al.* 2015). It has been suggested that brief high intensity activities are not always detected with a 1-min epoch length if the rest of the epoch consists of lighter activity (Trost *et al.* 2005; Michel & Brown 2011). In our study, a 1-min epoch length was used, which may have led to some unrecorded higher activity counts. In Michel and Brown (2011), the upper limit for sedentary activity count was 204 and the lower limit for high activity count 1,751, which are in line with our cut-off points (347 and 1,343, respectively). As in Michel and Brown (2011), for our study standardised activies (i.e. trotting and lying down) were used for cut-off point selection instead of spontaneous home environment activities. However, it remains uncertain how well these cut-off points differentiate movements of similar intensities (e.g. jumping, playing) in habitual physical activity.

Environment and owner-related factors are very important when evaluating pet dogs’ activity. Extreme weather conditions (i.e. hot, cold/icy and rainy weather) have been shown to have a negative impact on activity levels of dogs and their owners (Schneider *et al.* 2015; Aspvik *et al.* 2018; Wagner *et al.* 2019; Hall *et al.* 2021). Furthermore, as BOAS causes exercise intolerance and thermoregulatory disturbances, brachycephalic dogs are even more sensitive to temperature changes, and therefore, exercise might be proactively limited by the owner (Roedler *et al.* 2013; Aromaa *et al.* 2019; Packer *et al.* 2019). To diminish the impact of weather on individuals, measurements in our study were not collected during the summer months and owners were asked to avoid starting the measurement period during exceptional weather conditions. In our study, all dogs were family dogs whose activity has been shown to be mainly controlled by their owners (Griss et al. 2021). Dogs of physically active owners and with a stronger dog-owner relationship have been shown to be more active (Chan *et al.* 2005; Väätäjä *et al.* 2021). Additionally, owners are known to be motivated to increase their dogs’ physical activity during activity measurements (Zamansky *et al.* 2019). Dow *et al.* (2009) also demonstrated that companion dogs were more active during weekends and that a full seven-day measuring period gave rise to the most reliable estimate of total activity. Therefore, in our study we chose a seven-day activity monitoring with an owner diary to mimimise day-to-day variance and to more clearly evaluate dogs’ exercise habits and the living environment. We were not able to collect any owner-related personal data, such as family composition or owner age, and therefore these factors cannot be assessed in our study. However, in all study groups, no major differences emerged in owner questionnaire results regarding dogs’ health, living environment, exercise habits and deviations from weekly routines. Although orthopaedic and skin (i.e. pruritus) diseases can affect physical daily activity (Nuttall & McEwan 2006; Brown *et al.* 2010b; Wernimont *et al.* 2018) and are known to occur in brachycephalic breeds (O’Neill *et al.* 2016, 2018), all owners in our study considered their dogs to be in good health. Also those four dogs on atopy and one on epilepsy medication had their condition under control. However, the activity collar caused more discomfort in brachycephalic dogs than in controls. This might be explained by the fact that BOAS signs arise from the short anatomical structure of the neck and skull (Oechtering 2010; Emmerson 2014).

The duration of activity data collection per day has varied between studies, making comparisons difficult. A consistent finding of these studies has been that dogs spent most of their day in sedentary behaviour and the least time in high activity (Michel & Brown 2011; Morrison *et al.* 2013, 2014). In Michel and Brown’s study (2011), the median proportions of time dogs spent in different intensities of activity were 87% (range 65–95%) in sedentary, 11% (4–31%) in moderate and 2% (0–13%) in high. Additionally, Griss *et al.* (2021) demonstrated that family dogs were more often highly active and compensated with longer sedentary activity periods than free-roming dogs, which spent most of their time in moderate activity. The corresponding results of our study are similar, supporting that activity monitors can be used in dogs to distinguish standardised activities of different intensities. In our study, the total activity in BOAS+ French Bulldogs, BOAS– Pugs and BOAS+ Pugs was significantly lower than in control dogs. A closer assessment of differences in activity classes revealed that BOAS+ French Bulldogs spent significantly more time in sedentary activity and less time in high activity than control dogs. BOAS– Pugs and BOAS+ Pugs spent similarly significantly less time in high activity. Although control dogs had more opportunities to go outside unleashed than brachycephalic dogs, this is not a likely explanation of the higher activity seen in controls since a minority of high activity occurred during periods marked as free outside access. The limitation in both BOAS+ groups is the relatively low number of dogs with severe BOAS signs, and therefore, BOAS+ groups consisted mainly of dogs with moderate signs. As BOAS is dynamic by nature and stress/excitement can worsen the signs, dogs with mild (BOAS 1) or moderate (BOAS 2) grading can be relatively close to each other in clinical presentation. In Pugs, the BOAS– group consisted mainly of dogs with mild signs, in contrast to French Bulldogs, where the majority of BOAS– dogs had no signs at all (BOAS 0). This may partly explain why all Pugs appear to be more similar when comparing activity levels. In addition, since most BOAS– French Bulldogs had no clinically significant signs, as expected, their activity did not differ from control dogs.

It is important to acknowledge that body conformational factors, breed and age have an effect on the physical activity seen in pet dogs (Brown *et al.* 2010a; Morrison *et al.* 2013, 2014; Griss *et al.* 2021). Obesity has been shown to decrease physical activity in dogs (Chan *et al.* 2005; Warren *et al.* 2011; Morrison *et al.* 2013). Additionally, obesity can worsen signs of BOAS, i.e. compromise breathing and cause exercise and heat intolerance, which can affect dogs’ daily physical activity (German 2006; Manens *et al.* 2012; Packer *et al.* 2015). It has been previously shown that Pugs in particular tend to be overweight relative to other breeds (Such & German 2015; O’Neill *et al.* 2016; Liu *et al.* 2017; Aromaa *et al.* 2019). Unfortunately, in our study, body condition score was not available for analysis. Therefore, obesity can be one contributing factor to our activity results and might also partly explain similarity of the activity levels, especially between the BOAS– and BOAS+ Pug groups. However, the association between obesity and physical activity in dogs is likely to be as complex as in humans, warranting further investigations (Davis *et al.* 2006; Hughes *et al.* 2006, 2008; Ekelund *et al.* 2008; Councier *et al.* 2010; Morrison *et al.* 2013). Breed must also be considered since sedentary behaviour has been shown to be significantly higher in Labrador Retrievers than in Cocker Spaniels (Morrison *et al.* 2014). To diminish the influence of breed on activity measurements, the brachycephalic dogs in our study were analysed in breed groups, not together. In addition, the control group consisted of size-matched, non-brachycephalic dogs of different breeds to minimise the impact of a single breed variable.

As in humans, dogs’ physical activity has been shown to decrease with age (Sallis 2000; Troiano *et al.* 2008; Michel & Brown 2011; Zanghi *et al.* 2012; Morrison *et al.* 2014). In two companion animal studies (Michel & Brown 2011; Morrison *et al.* 2014), moderate and high activity were significantly decreased and sedentary behaviour increased as dogs aged. In our study, a significant effect of ageing was seen in controls and Pugs in all activity classes. Comparable human studies mainly focus on one age group at a time, which diminishes the explanatory effect of ageing on activity measurements (Schmitz *et al.* 2002; Trost *et al.* 2002; King *et al.* 2011). Morrison *et al.* (2014) discussed in their study that wider age distribution could be one reason why ageing seems to be a more powerful correlate. When reviewing the age distribution of our study groups, French Bulldogs are of a narrower age distribution than Pugs and controls, supporting this hypothesis.

### Animal welfare implications

Our study population mainly comprised dogs in good health and only a few had severe signs of BOAS. In both bracycephalic breeds, the BOAS+ dogs were less active than controls, and in Pugs this difference was also seen with BOAS– dogs. Although age affects the results and Pugs were somewhat older than French Bulldogs and controls, our findings indicate that BOAS decreases habitual physical activity, thereby impacting dogs’ QOL even in less-affected dogs. However, it is an encouraging finding that the activity of BOAS– French Bulldogs, consisting mainly of asymptomatic brachycephalic dogs, is not significantly different from that of same-aged control dogs. Our results further support the importance of actions taken against the harmful impacts of BOAS such as breeding tests (Lilja-Maula *et al.* 2017; Aromaa *et al.* 2019; Riggs *et al.* 2019) and raising public awareness.

## Conclusion

Our results show that brachycephalic dogs with more severe BOAS signs are less active in everyday life than non-brachycephalic control dogs when assessed with an activity monitor. Our study further confirms that accelerometer-based activity monitoring is easy to use and a well-tolerated method to assess habitual physical activity in pet dogs, although the activity collar can cause more discomfort in brachycephalic dogs. Age is known to decrease physical activity, and this was seen in our study in controls and Pugs, both of which showed a wider age distribution than French Bulldogs. As BOAS signs can worsen with age, it is important to examine the differences in daily physical activity in even more strictly chosen weight and age groups, and to compare more senior brachycephalic dogs with similarly aged controls.

## Supplementary material

For supplementary material accompanying this paper visit https://doi.org/10.1017/awf.2023.80. click here to view supplementary material
