# Supplementary material for: Comparison of habitual physical activity in French Bulldogs, Pugs and normocephalic dogs by accelerometry
Source: Anim Welf. 2023 Sep 11;32:e60. doi: 10.1017/awf.2023.80 (PMC10936329; doi:10.1017/awf.2023.80)

Supplementary figure 1 Box plot of mean daily activity counts in 1.A brachycephalic obstructive airway syndrome (BOAS) positive (+) and negative (-) French Bulldogs (FB) and 1.B Pugs compared with controls with median +/- IQR after exclusion of one control dog (red square). Four dogs that had most opportunities to go outside unleashed are highlighted in red. Significant differences (P <0.05) between groups are marked.

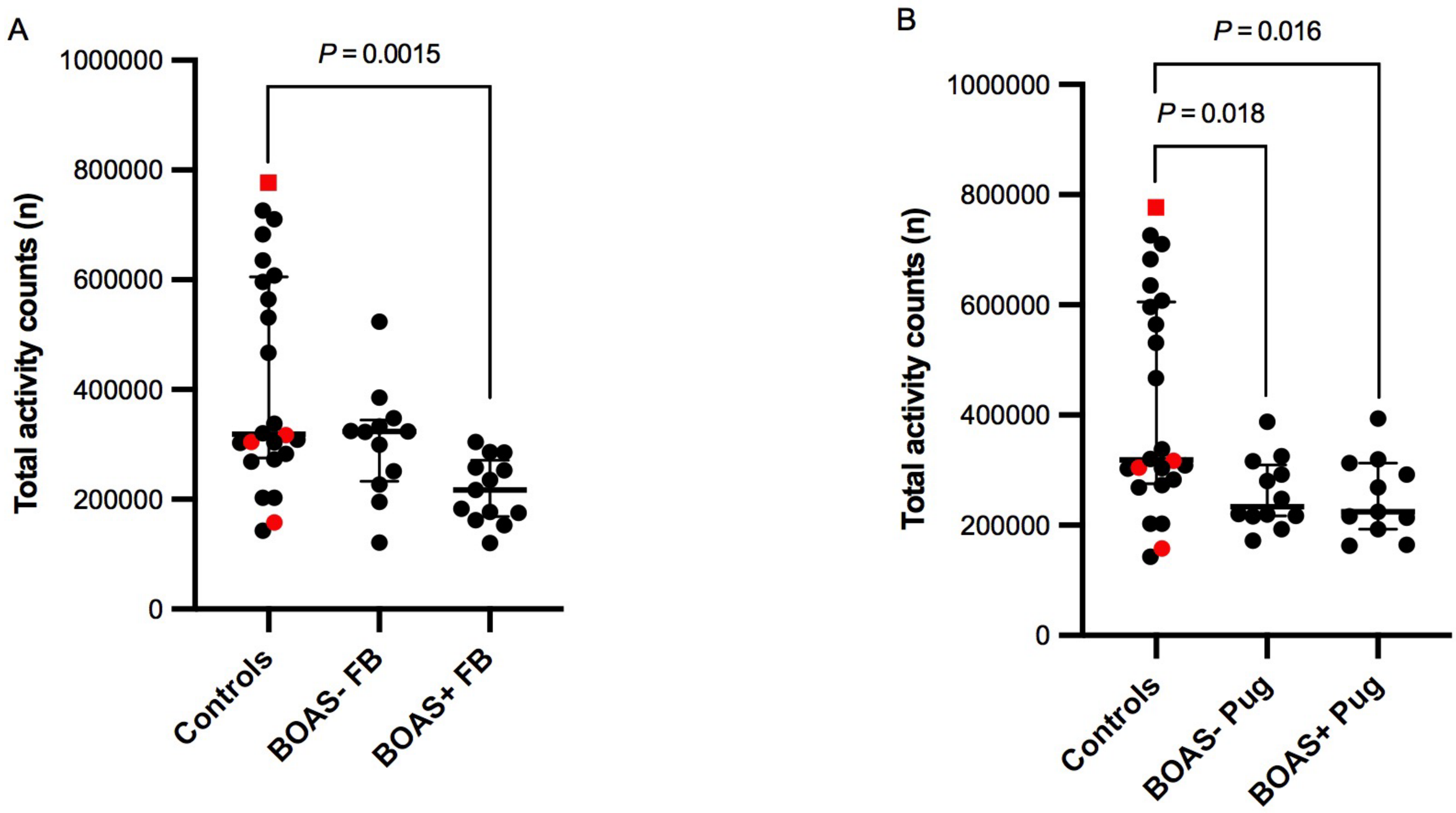

Supplement: Supplementary file 1 [file awfsup.zip › S0962728623000805sup001.pdf]
