# Supplementary material for: Comparison of habitual physical activity in French Bulldogs, Pugs and normocephalic dogs by accelerometry
Source: Anim Welf. 2023 Sep 11;32:e60. doi: 10.1017/awf.2023.80 (PMC10936329; doi:10.1017/awf.2023.80)

Supplementary figure 2 The distribution of weight in different sex and age groups in relation to total activity count in French Bulldogs (FBs) and Pugs

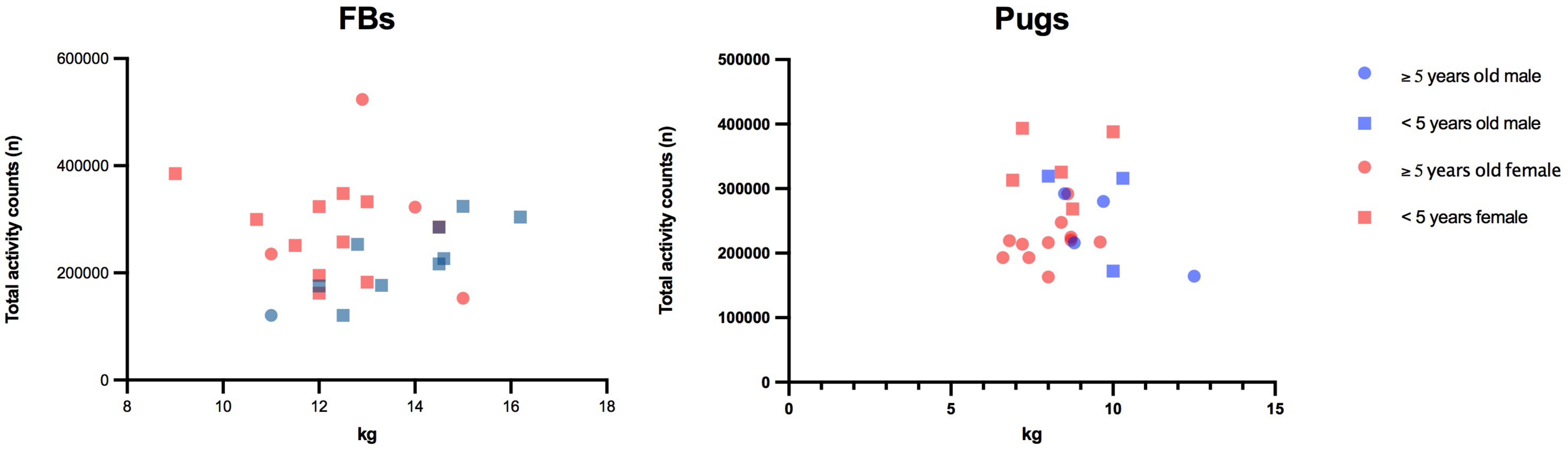

Supplement: Supplementary file 1 [file awfsup.zip › S0962728623000805sup002.pdf]
