# Supplementary material for: Comparison of habitual physical activity in French Bulldogs, Pugs and normocephalic dogs by accelerometry
Source: Anim Welf. 2023 Sep 11;32:e60. doi: 10.1017/awf.2023.80 (PMC10936329; doi:10.1017/awf.2023.80)
Supplement: Supplementary file 1 [file awfsup.zip › S0962728623000805sup003.pdf]

Questionnaire for the activity study

Owner____

Email address_____________________________________

Phone____

Address____

Dog´s name_____________________

Sex______________

Birth date_____________________

Weight_______________________

The collar was worn for the seven-day period starting from

Time _____ day ______/______/20__

The collar was removed

Time_____day _____/_____/20__

Activity collar

Was the seven-day period a typical week for your dog? NO/YES

If not, what was atypical comparing to your normal routines?

____

____

Was the collar disturbing your dog? NO/YES

If yes in which situations did your dog react to the collar and how?

____

____

Did you remove the collar during the seven-day measurement days? NO/YES

If yes in which situations and why

____
____

The study week

Overall health status during the seven-day period:

Appetite: Normal ☐ Decreased ☐ Increased ☐

Drinking: Normal ☐ Decreased ☐ Increased ☐

Urinating: Normal ☐ Abnormal ☐

Defecating: Normal ☐ Abnormal ☐

Did your dog vomit/regurgitate during the seven-day measurement period? NO/YES

If yes, describe and in what kind of situations?

____
____

Did your dog have any pruritus or skin/ear disease during the seven-day measurement period? NO/YES

If yes, describe and in what kind of situations?

____

____

Was there coughing during the seven-say measurement period? NO/YES

If Yes, describe

____
____

<u>Basic information on your dog´s health</u>

Has your dog had any surgical operations? NO/YES

If Yes, what and when?

____

Does your dog have any restrictions or medical conditions affecting its overall condition or daily living? NO/YES

If yes, describe____

If yes, are the signs present all the time? NO/YES

If no, when?____

Is your dog on any medical treatment currently? NO/YES

If Yes, why/what? ____

Does your dog have exercise intolerance in general? NO/YES

If Yes, describe

____

____

If Yes, is it worse with warm temperatures: NO/YES

Does your dog have loud breathing sounds/snoring (circle one or multiple)?

In rest, in exercise, in warm/very cold weather, when sleeping, never

Has your dog ever shown cyanosis (= blue discoloration of mucous membranes)? YES/NO

If Yes, when and how often? ____

Does your dog have choking fits? YES/NO

If Yes Daily, Weekly, Occasionally

If Yes describe in what situations?____

Has your dog ever fainted? YES/NO

If Yes, when and how often?____

In what position does your dog usually sleep (circle one)?

Head at the level of body, Head elevated, Sitting position, Toy in mouth

Are there other animals in the household? YES/NO

If yes, what? ____

Outdoor habits in everyday life

My dog takes walks with me on a leash

Never ☐ Sometimes ☐ Often ☐ Always ☐

My dog takes walks with me unleashed

Never ☐ Sometimes ☐ Often ☐ Always ☐

My dog has a free access to go outside on its own

Never ☐ Sometimes ☐ Often ☐ Always ☐

Please describe in more detail your dog´s outdoor habits in everyday life if previously mentioned options do not give a correct/accurate description of your dog´s daily exercise:

____

____

____

____

____

____

____

____

____

____
