# Supplementary material for: Comparison of habitual physical activity in French Bulldogs, Pugs and normocephalic dogs by accelerometry
Source: Anim Welf. 2023 Sep 11;32:e60. doi: 10.1017/awf.2023.80 (PMC10936329; doi:10.1017/awf.2023.80)
Supplement: Supplementary file 1 [file awfsup.zip › S0962728623000805sup004.pdf]

EXAMPLE

The activity diary

12:00 ap.

TIME INTERVAL 30 MIN

›

AIKA	1. Day	2. Day	3. Day	4. Day	5. Day	6. Day	7. Day
12:00 AM							
12:30 AM							
1:00 AM							
1:30 AM							
2:00 AM							
2:30 AM	Sleeping						
3:00 AM							
3:30 AM							
4:00 AM							
4:30 AM							
5:00 AM							
5:30 AM							
6:00 AM							
6:30 AM							
7:00 AM							
7:30 AM							
8:00 AM							
8:30 AM	walked						
9:00 AM							
9:30 AM							
10:00 AM							
10:30 AM							
11:00 AM	home						
11:30 AM	alone						
12:00 PM							
12:30 PM							
1:00 PM							
1:30 PM							
2:00 PM							
2:30 PM							
3:00 PM							
3:30 PM							
4:00 PM							
4:30 PM							
5:00 PM							
5:30 PM	walked						
6:00 PM							
6:30 PM							

AIKA	1. Day	2. Day	3. Day	4. Day	5. Day	6. Day	7. Day
7:00 PM	Walked						
7:30 PM							
8:00 PM							
8:30 PM							
**9:00 PM**	at						
9:30 PM	home						
10:00 PM							
10:30 PM							
11:00 PM							
11:30 PM	Sleeping						
